# InFusion: Advancing Discovery of Fusion Genes and Chimeric Transcripts from Deep RNA-Sequencing Data

**DOI:** 10.1371/journal.pone.0167417

**Published:** 2016-12-01

**Authors:** Konstantin Okonechnikov, Aki Imai-Matsushima, Lukas Paul, Alexander Seitz, Thomas F. Meyer, Fernando Garcia-Alcalde

**Affiliations:** 1 Department of Molecular Biology, Max Planck Institute for Infection Biology, Berlin, Germany; 2 Lexogen GmbH, Campus Vienna Biocenter 5, Vienna, Austria; Universita degli Studi di Torino, ITALY

## Abstract

Analysis of fusion transcripts has become increasingly important due to their link with cancer development. Since high-throughput sequencing approaches survey fusion events exhaustively, several computational methods for the detection of gene fusions from RNA-seq data have been developed. This kind of analysis, however, is complicated by native trans-splicing events, the splicing-induced complexity of the transcriptome and biases and artefacts introduced in experiments and data analysis. There are a number of tools available for the detection of fusions from RNA-seq data; however, certain differences in specificity and sensitivity between commonly used approaches have been found. The ability to detect gene fusions of different types, including isoform fusions and fusions involving non-coding regions, has not been thoroughly studied yet. Here, we propose a novel computational toolkit called InFusion for fusion gene detection from RNA-seq data. InFusion introduces several unique features, such as discovery of fusions involving intergenic regions, and detection of anti-sense transcription in chimeric RNAs based on strand-specificity. Our approach demonstrates superior detection accuracy on simulated data and several public RNA-seq datasets. This improved performance was also evident when evaluating data from RNA deep-sequencing of two well-established prostate cancer cell lines. InFusion identified 26 novel fusion events that were validated in vitro, including alternatively spliced gene fusion isoforms and chimeric transcripts that include intergenic regions. The toolkit is freely available to download from http:/bitbucket.org/kokonech/infusion.

## Introduction

Since the discovery of the BCR–ABL1 fusion gene and its critical role in the development of chronic myeloid leukemia [1] the search for gene fusions has attracted a great amount of interest among cancer researchers. Gene fusions, which result from genomic translocations, inversions or deletions, may be closely related to cancer progression and play a driver role in particular types of cancers [2]. Examples include the TMPRSS2–ERG fusion in a large family of prostate cancers [3], EML4–ALK in non-small-cell lung cancer [4] and ETV–NTRK3 in several different cancers [5, 6]. Chimeric transcripts, which are composed from exons of different genes, can also occur in normal cells due to trans-splicing events [7], transcriptional slippage [8] or errors of the transcription machinery such as gene read-through [9], and in certain cases they have been reported to be functional in mammalian genomes [10]. While the recent progress of sequencing technologies has enabled the genome- and transcriptome-wide study of chimeric RNAs with unprecedented depth and sensitivity, there is a need to develop and apply reliable and efficient computational methods for gene fusion and chimeric transcript detection from sequencing data. As well as furthering basic cancer research, the discovery of gene fusions by means of deep sequencing is also expected to play a useful role in personalized medicine and targeted therapy in the future.

High throughput RNA sequencing (RNA-seq) provides an effective way to study the transcriptome [11]. Despite the smaller size of the transcriptome with respect to the genome, RNA-seq data analysis is challenged by the existence of complex regulatory mechanisms like alternative splicing, transcription of processed pseudogenes, dynamic concentration range of isoform expression, etc. High-throughput sequencing also suffers from inherent difficulties such as PCR-amplification bias, uneven fragment distribution [12, 13] and generation of false chimeras due to template-switching during reverse transcription errors [14]. Thus, rigorous quality control measures and advanced bioinformatics algorithms are required for processing RNA-seq data [15–17].

Studying gene fusions in the transcriptome allows detection of rearrangements that might be translated into novel functional proteins. Maher et al. [18] were one of the first to apply RNA-seq for gene fusion discovery in several cancer cell lines. They were able to not only confirm previously described fusions and chimeric transcripts, but also detect and validate a number of novel events. After these pioneering studies demonstrated the suitability of the approach, efforts intensified to develop efficient computational methods for the detection of fusion genes from RNA-seq data.

FusionSeq was one of the first published computational pipelines for fusion gene discovery from RNA-seq data [19]. The method is based on the detection of discordantly aligned read pairs, which are used to construct a junction library of possible fused exon pairs. The sequencing reads are then realigned to this constructed library to find the exact fusion junctions. Although the authors established the basis for organizing a pipeline for fusion gene detection, practical application of FusionSeq on a variety of datasets revealed that it is not equally sensitive in all cases [20]. Other methods such as TopHat-Fusion [21] and ChimeraScan [22] are based on detecting reads that cover the junction of genes involved in a putative fusion event (so-called SPLIT-read approach, see Materials and Methods for more details). However, due to the small size of the sequencing reads and the repetitive nature of the genome, this approach requires intensive filtering to remove the large number of false positives.

An advanced computational method, deFuse [23], employs discordantly aligned pairs (so-called BRIDGE reads) for initial fusion discovery, followed by the application of the SPLIT-read approach to find the exact fusion breakpoint location. It improves specificity of the discovery by utilizing machine learning techniques to better distinguish between true and false positive predictions. There are also several novel methods such as SOAPfuse [24] and fusionCatcher [25] that advance various aspects of fusion gene discovery, e.g. fusion isoform detection, prediction accuracy and computational resource usage. Additional attempts have been made to discover fusions using reference-guided assembly of chimeric transcripts [26, 27]. A major drawback of such an approach is that it relies on detection of possible discordant read alignments and also requires as much filtering as SPLIT-read based methods

Although a number of state-of-the-art methods are currently available, reliable discovery of the whole spectrum of different fusion events from RNA-seq data is a challenging task [28] and, to our knowledge, a systematic assessment of the ability to report various fusion classes such as genomic rearrangements [2] or trans- and cis-splicing events [29] among available methods has not been performed so far. In addition, a recent analysis [30], as well as our own investigations revealed differences in results between commonly used methods applied to the same validated datasets. An additional complexity is presented by antisense transcription, which can also occur in fusions [31]. Up to now, only TopHat-Fusion and ChimeraScan take the strand specificity provided by the library preparation protocol into account, but they do not mark whether a detected fusion was transcribed in the sense or antisense direction. Additionally, there is occasional evidence of functional fusion events with a breakpoint inside an exon [32] or involving non-coding [33], intronic [34, 35] or even intergenic regions [36]. Several studies have reported alternatively spliced isoforms of fusion genes [34, 37, 38]. Certain existing tools, including TopHat-Fusion, deFuse, SOAPfuse and fusionCatcher, are capable of detecting fusion isoforms. Likewise, deFuse and TopHat-Fusion can also discover fusions that involve intronic regions.

In this work we describe InFusion, a novel computational method for the discovery of chimeric transcripts from RNA-seq data capable of detecting alternatively spliced chimeric transcripts and fusion genes involving non-coding regions. Specifically, InFusion allows detection of fusions that involve intergenic regions, which to our knowledge has not been addressed previously. The method applies a novel algorithmic approach to cluster and reconstruct fusions from SPLIT and BRIDGE reads. Additionally, it analyzes and filters putative fusion events based on coverage depth, genomic context and strand specificity. We found that InFusion shows improved accuracy on simulated and a number of public datasets. We experimentally validated our method by performing strand-specific deep RNA-sequencing of two well-characterized prostate cancer cell lines. Overall, InFusion discovered more than 400 fusions for each cell line (from ~ 80M RNA-seq reads each), and from 40 tested fusions we confirmed 10 known and 26 previously unreported fusion transcripts using qRT-PCR. Among these validated transcripts are several novel alternatively spliced isoforms of well-known fusions and some that involve fusion of an intergenic region with a coding one.

The InFusion pipeline was developed in C++ and Python. It is capable of working with both single-end and paired-end sequencing reads. It is free for academic use and can be downloaded from https://bitbucket.org/kokonech/infusion.

## Materials and Methods

### InFusion Pipeline

The InFusion pipeline consists of several independent steps, each of which is controlled by a number of configuration parameters. The typical input for the pipeline is a set of single-end or paired-end RNA-seq reads. Most of the pipeline components are implemented in C++ using the SeqAn bioinformatics library for efficient computations [39]. InFusion relies on the genome and transcriptome information from the organism of interest in an indexed format. The pipeline provides the functionality to automatically download required annotation and sequence files and perform indexing of genome and transcriptome sequences.

**Fig 1:**
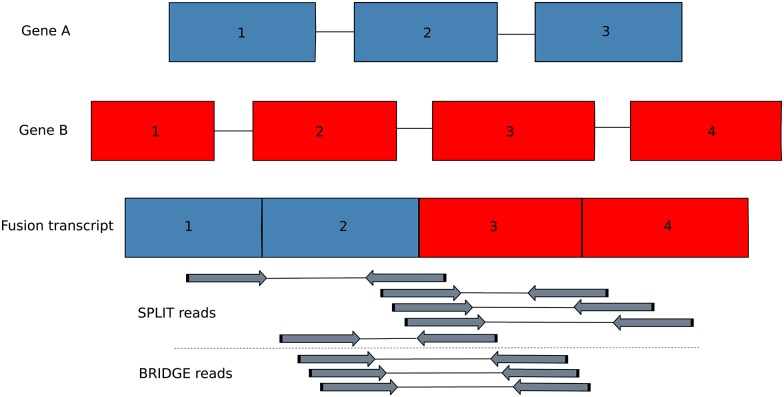
Example of fusion detection from RNA-seq data. The fusion consists of exons 1 and 2 from gene A and exons 3 and 4 from gene B. SPLIT reads cover the junction point, while BRIDGE reads span the junction point within the insert region, which is not sequenced.

Our algorithm is based on detection of two basic events that allow gene fusion detection from sequencing reads. These events can be demonstrated using a hypothetical fusion of two genes (Fig 1). The first event occurs when the read spans the fusion junction, termed a SPLIT read. The second event requires the reads to be paired-end. In this case, a pair of reads from the same fragment spans the fusion within the non-sequenced part of the insert, termed a BRIDGE read pair.

**Fig 2:**
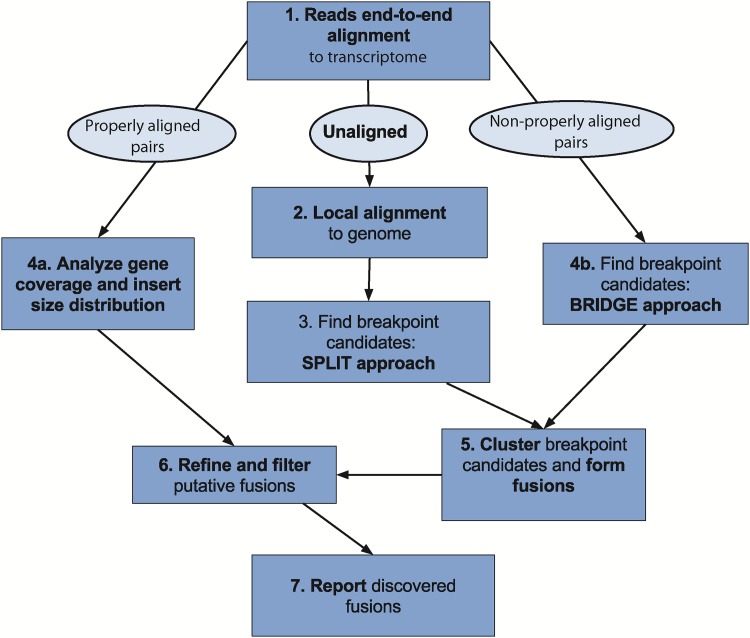
InFusion pipeline overview. Outline of the different steps of the analysis pipeline. Properly aligned pairs require the distance between alignments to be within the maximum intron size on the same chromosome.

The InFusion analysis pipeline is outlined in Fig 2. The pipeline starts with the mapping of reads to the transcriptome and, optionally, to the genome (Step 1), while keeping track of the unmapped reads. The unmapped reads from the previous step are aligned locally to the genome (Step 2). The resulting local alignments are used to detect potential SPLIT reads (Step 3). Next, the reads aligned to transcriptome and genome are analyzed to collect insert size statistics and detect discordantly mapped read pairs that form BRIDGE read pairs (Step 4). This step is skipped for single-end sequencing experiments. Next, SPLIT and BRIDGE reads that potentially belong to the same fusion are clustered (Step 5). During this step, the pipeline tries to rescue SPLIT reads that were not detected during initial analysis of local alignments. Finally, putative fusions are analyzed to filter false positive events (Step 6), and discovered chimeric transcripts are reported (Step 7). The first four analysis steps were introduced previously and are used by tools such as chimeraScan, deFuse, SOAPfuse and others. However, InFusion provides several improvements to these steps, and there are a number of novelties in the following analysis procedure: clustering of detected SPLIT and BRIDGE reads, advanced filtering and recovery along with special statistics computation for fusion reports. Below, each step of the pipeline is described in detail.

### Alignment of Short Reads

The main task of the read alignment is to find short reads that could reveal putative fusion events and separate them from those originating from “normal” genes. In addition, the alignment of reads is used to collect statistical information about the sequencing experiment, such as insert size distribution and gene expression levels, which is used later in the analysis and filtering of putative fusion events. Firstly, the given short-reads are mapped to the transcriptome. To reduce the pipeline running time, reads that were not mapped to the transcriptome can optionally be mapped to the genome, since there could be reads that are only fully mapped to the genome. Additionally, by default the reads mapped to the mitochondria are discarded from further analysis. For the alignment of the reads InFusion uses Bowtie2 [40] by default, however, any modern short read aligner could potentially be used.

### Local Alignment of the Short Reads

In order to search for possible SPLIT candidates, unmapped reads reported during the initial alignment are further aligned locally to the genome. Local alignment of short reads to the genome is performed using Bowtie2 in local mode. Similar to the previous step, InFusion can potentially be used with other aligners that support local mapping. The most crucial parameter of the local alignment is the minimal score to consider a valid mapping. InFusion is designed to accept mappings with a score greater than $\frac{Score_{max}}{3}$, where *Score*_*max*_ denotes the maximum alignment score within a given scoring scheme. The following scoring scheme is used by default: 2 for match, -2 for mismatch, -6 for gap open, -3 for gap extension.

### Analysis of Local Alignments

Each local alignment of a read is analyzed to detect if it is possible to form a SPLIT candidate from it. We define the following conditions. Let us assume that there are *n* local alignments of a read:

$$\left\{A_{1},A_{2},\ldots ,A_{n}\right\}$$

Each alignment *A*_*i*_ is represented by reference sequence (chromosome) *c*_*i*_, starting position in the reference sequence coordinates *p*_*i*_, starting position in the read coordinates *q*_*i*_ and length *l*_*i*_:

$$A_{i}:\left\{c_{i},p_{i},q_{i},l_{i}\right\}$$

We consider that 2 local alignments *A*_*i*_ and *A*_*j*_ form a SPLIT read if two conditions apply:

1. Either *c*_*i*_ ≠ *c*_*j*_ or *p*_*i*_ + *l*_*i*_ − *p*_*j*_ > *l*_*max*_

The alignments are coming from different chromosomes or the distance between alignments is larger than maximum intron size. Maximum intron size *I*_*max*_ depends on the genome studied. The default value refers to the human genome with a maximum intron size of 20,000.

2. *q*_*i*_ < *q*_*j*_ *and* (−1) × *T*_*outer*_ < *q*_*j*_ − (*q*_*j*_ + *l*_*i*_) < *T*_*inner*_

The alignments are concordant in the read coordinates based on given thresholds: maximum intersection size *T*_*inner*_ and maximum distance between alignments *T*_*outer*_ in the coordinates of the read. By default we set these thresholds to 10 and 2 bp, respectively.

To increase the sensitivity of discovery we allow multiple local alignments of a single read. The multimappings are resolved in later steps of the pipeline. InFusion accounts only for SPLIT reads which are formed by two local mappings and by default allows up to 20 possible SPLIT configurations to be formed from the same read.

### Analysis of End-to-end Alignments

Paired-end sequencing experiments make it possible to search for BRIDGE reads. To perform this task, we analyze the not-correctly aligned read pairs from Step 1. To detect not-correctly aligned read pairs firstly, the transcriptomic alignments are converted into genomic coordinates. Two mate alignments *M*_*1*_ and *M*_*2*_ form a correct pair if they are aligned on the same chromosome within a distance of the defined maximum intron size *I*_*max*_, otherwise they form a possible BRIDGE read pair. We record all discovered BRIDGE read pairs for further analysis. In addition, we compute the insert size distribution from concordant alignments and estimate the expression level for each gene.

### Clustering and Forming Putative Fusions

A chimeric transcript can be described by a pair of genomic coordinates, which represent the fusion breakpoint. For example, the fusion gene shown in Fig 1 has a breakpoint formed by the last base pair of exon 2 of gene A and the first base pair of exon 3 of gene B. We refer to the SPLIT reads and BRIDGE reads detected in previous steps as breakpoint candidates (BPCs). It should be noted that a SPLIT read implies an exact breakpoint, while a BRIDGE pair implies an approximate breakpoint within the corresponding insert size distance. Thus, each BPC is described by two alignments, which are the local alignments of the read if the BPC is formed by a SPLIT read, and the end-to-end alignments of a pair of reads if the BPC is formed by a BRIGDE pair.

A clustering procedure groups the alignments forming the BPCs into clusters based on their genomic coordinates. Thus, alignments of each BPC are iteratively analyzed: *i)* if there is no intersection with other existing clusters the alignment constitutes a new cluster; *ii)* if the alignment intersects with one existing cluster, it is added to this cluster; *iii)* if the alignment belongs to two or more clusters simultaneously, the clusters are first merged into one single cluster and the alignment is then added to it.

**Fig 3:**
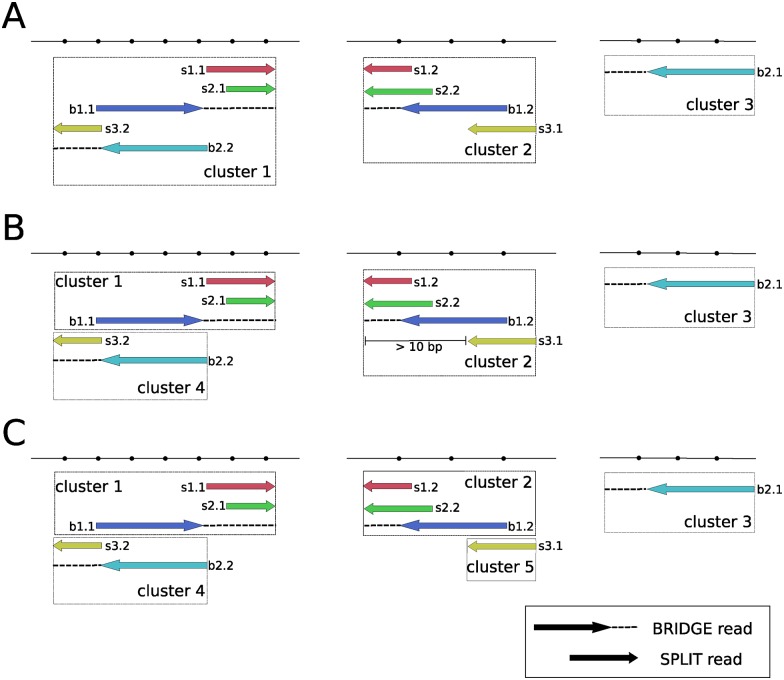
Clustering of breakpoint candidates. The arrows of the SPLIT alignments and the dot lines of BRIDGE alignments demonstrate the direction to the breakpoint position. (A) Initial clusters are created from intersecting SPLIT and BRIDGE alignments. (B) Cluster 4 is separated from cluster 1 based on the directionality, which is inferred from the alignment strand and order. (C) Cluster 5 is separated from cluster 2 based on the putative breakpoint position. Alignments belonging to the same breakpoint candidate have the same color. BRIDGE reads are marked with b, SPLIT reads are marked with s. A SPLIT read assumes an exact breakpoint, while a BRIDGE read assumes an approximate breakpoint within allowed insert size distance.

As a result of the initial clustering procedure (Fig 3A), clusters can contain multiple possible breakpoints represented by different groups of alignments. Therefore, we further separate the clusters based on their directionality: the alignment strand and the order of the alignment in the BPC, defined by location in the read coordinates in case of a SPLIT read or mate identifier in case of a BRIDGE read, predict the direction to the breakpoint position, situated either upstream (5’-to-3’) or downstream (3’-to-5’) of it (Fig 3B).

We next analyze if the coordinates of the breakpoint positions implied by local alignments in a cluster are compatible within a configurable tolerance (10 bp by default). If this is not the case, the cluster is separated into 2 new clusters. (Fig 3C) The process continues until there are no more clusters with significant difference in the coordinates of the breakpoint position left. We further refine the clusters by assigning to them the compatible unused local alignments of reads from Step 2 of the pipeline that have an alignment score greater than 50% of the maximum score and an edit distance less than 2 (both options are configurable). Using an interval tree data structure, we intersect the read alignments with the existing clusters. For each intersecting cluster (which we call host cluster) found, we make sure that the alignment is concordant with the putative breakpoint position as it is dictated by the directionality and alignments from SPLIT reads in the cluster. We then select the remaining unmapped part of the read sequence and try to realign it to each potential fusion partner cluster of the host cluster, again in concordance with cluster directionality and the fusion breakpoint.

Clusters formed solely from BRIDGE pairs constitute a special case in the rescue procedure described above. For this type of cluster the exact breakpoint position cannot be computed, therefore we allow additional tolerance (computed from insert size distribution) in the genomic region upstream to the breakpoint position as dictated by the pair configuration. The rescued reads found in this case undergo an additional clean-up procedure, which selects the most probable breakpoint based on the amount of evidence for a particular position.

Finally we go through the list of BPCs to form putative fusions. For every BPC we check to which cluster each of its alignments belongs. Then we assign the BPC to a putative fusion event described by two unique cluster identifiers. During the creation of putative fusions we also take into account the strand-specificity of the sequencing library to reconstruct the correct 5’-3’ order of the fusion transcript. If the sequencing protocol is non-strand-specific, the information from the annotation is applied to reveal the possible order of genes forming the fusion.

### Refining and Filtering Fusions

In our investigations we observed that the majority of validated fusion genes discovered from paired-end RNA sequencing data are supported by SPLIT reads with a correct mate pair connection. Because of this, we make use of the paired-end information by discarding SPLIT reads from putative fusions which do not have their mate-pair located within the maximum intron size. Additionally, clusters which consist uniquely of BRIDGE reads are merged with compatible clusters that are located within maximum intron size in order to avoid reporting two fusions associated with the same event.

We resolve multimapped reads by iteratively assigning each read with several mappings to one putative fusion with the largest score among other fusions, similar to the deFuse algorithm. The fusion score is a function of the number of supporting reads calculated by taking into account their alignment type, multi-mapping status and the presence of a mate pair for BPCs originating from SPLIT reads. The types of supporting read filters are described in S1 Text.

After resolving multimapped reads, we further calculate features associated with each fusion that are used to filter and analyze the fusion:

#### Metacluster homogeneity

By analyzing the genomic intervals forming the putative fusion, we noticed that clusters of false positive fusions arising from local homology (sequence similarity) are usually found intersecting or close to each other in a small region. By contrast, cluster blocks forming the true fusions usually dominate over other clusters in the selected region i.e. the majority of breakpoint candidates in a region belong to the same cluster block. We defined intersecting cluster blocks belonging to different fusions as a metacluster. Avoiding false positive fusions reported due to homology is possible by filtering SPLIT-read and BRIDGE-read alignments from repeat regions. However, if the repeat regions are not provided for the genome or not detailed enough, then it is important to perform metacluster homogeneity analysis. We apply this observation by calculating the number of supporting alignments for each fusion cluster divided by the total number of supporting alignments in the metacluster and filtering out those fusions that have low weight.

#### Read coverage

We reconstruct the sequence formed from detected fusion regions and calculate the coverage of the supporting SPLIT reads. For each fusion we compute the proportion of unique alignments based on their genomic coordinates and estimate if the mean breakpoint position in the read coordinates follows a uniform distribution. The predicted fusion is filtered if the proportion of unique SPLIT read alignments is close to zero or the mean breakpoint position inside the reads deviates significantly from the middle of the read. Additionally, if the fusion is supported only with SPLIT reads, we require at least one SPLIT read that does not have any multimappings in order to accept the predicted event. Moreover, correctly aligned reads coverage close to breakpoint is analyzed to detect if the transcripts forming the fusion are also expressed.

#### Homology

We construct the fusion sequence bounded by the read evidence and align it to the genome and transcriptome. By examining the alignment score, we can filter out false positive predictions arising from highly homologous genes.

#### Insert size

The reconstructed fusion is used to calculate the insert size for each of the supporting BRIDGE reads. The insert size is considered valid if it lies within a 3σ interval as defined by the insert size distribution, computed in Step 4a. The ratio of valid insert sizes is calculated and used as filtering parameter.

#### Strand specificity

Strand-specific protocols are preferable for fusion discovery since they make it possible to detect antisense transcription in fusions and infer the direction of transcription in fusions involving unannotated and intergenic regions. If the strand specificity is enabled, we calculate the proportion of supporting reads which are aligned according to the protocol and annotated strand of the gene. The computed metrics allow analysis of antisense transcription in the fusion.

#### Biotypes control

The selection of types of fusions to report, e.g. formed from intergenic regions or non-coding RNA, can be controlled through the settings. Additionally, by default fusions formed from pseudogenes and processed transcripts are discarded.

#### Final filtering

The final filtering of fusions is performed by applying configurable thresholds to the computed features. Default thresholds aim to provide a compromise between recall and precision, based on our experience with analyzing human RNA-seq datasets. Additionally, InFusion allows repeated filtering of fusions with adjusted thresholds without running the whole pipeline again. Decreasing most of the default filtering limits such as minimum number of supporting reads or homogeneity weight will increase the recall, while the reverse strategy will increase the precision.

### Reporting Fusions

For each predicted fusion event, InFusion reports the corresponding genomic regions, the coordinates of the breakpoint, as well as the number of supporting SPLIT and BRIDGE reads together with the features computed during putative fusion analysis and used in the filtering process. Additionally, the genomic regions involved are characterized by annotating implicated transcripts/exons, determining the possible type of fusion event, e.g. inter-chromosomal [2] or read-through [9]. Optionally, InFusion reports the fusion junction sequence, which can be useful for PCR primer design, as well as the original alignments of reads supporting the predicted events in BAM format.

### Simulation Pipeline

We developed a fusion gene simulation pipeline that enables construction of fusion gene annotations with given properties. We defined 5 classes of different fusion events:

1. With both fusion partners having a breakpoint at the exon boundary;
2. With one or both fusion partner(s) having a breakpoint inside an exon;
3. With one or both fusion partner(s) having a breakpoint inside an intron;
4. With one fusion partner originating from an intergenic region;
5. With several alternatively spliced isoforms having breakpoints at the exon boundary.

The pipeline uses gene annotations and genome sequence as input. It is designed to make every run reproducible and is available as part of the InFusion source code package. Using this pipeline, it is possible to create a required number of random fusion gene pairs of a selected class.

For our simulation experiments we generated 50 sets of fusion annotations, each consisting of 100 fusion events (20 of every predefined class). Next, for each set of the generated annotations describing the fusions we recreated transcript sequences and randomly assigned coverage ranging from 1X to 60X. Using the Mason software package [41], we simulated paired-end sequencing reads from these transcripts based on the assigned coverage in a non-strand-specific manner. For read simulation we used the Illumina error model, which includes mismatches, insertions and deletions. The length of the read was chosen as 75 bp with a mean fragment length of 300 bp and a standard deviation of 80 bp. Overall we generated 50 RNA-seq datasets containing evidence for 5,000 fusion genes. Additionally, using read counts from a real RNA-seq experiment [42] and the same Mason settings as for the fusion transcripts, we generated approximately 30M background reads. These background reads were added to each simulated dataset.

The simulation pipeline is available as a block of the InFusion toolkit. It allows configuration of options such as read size, insert size and strand-specificity. More information about the simulation pipeline is provided in S1 Text.

### Public Datasets

Three public RNA-seq datasets from cancer studies were used to test the performance of the InFusion pipeline: Edgren et al. [42] describe the transcriptome of cells originating from breast cancer, Berger et al. [43] from melanoma and Wu et al. [44] from prostate cancer. Each study provides evidence for fusion genes that were first detected from RNA-seq data and then experimentally validated using RT-PCR or Sanger sequencing.

### Cell Cultures and RNA Extraction

For experimental validation we used the prostate cancer cell line VCaP and the normal prostate cell line RWPE-1, which were obtained from the American Type Culture Collection (ATCC). VCaP was cultured in Dulbecco’s modified Eagle's medium (Life Technologies, Darmstadt, Germany) supplemented with 2 mM L-glutamine, 1 mM sodium pyruvate, 10% fetal calf serum (FCS, Biochrom, Berlin, Germany), and RWPE-1 in Keratinocyte serum-free medium supplemented with 5 ng/mL EGF and 50 μg/mL bovine pituitary extract (Life Technologies). An additional prostate cancer cell line, LNCaP, was provided by Prof. Klaus Jung (Department of Urology, Charité University Hospital, Berlin, Germany) and cultured in RPMI1640 (Life Technologies) supplemented with 10% FCS. PrEC (Lonza, Basel, Switzerland) was cultured in PrEGM bullet kit (Lonza), according to the supplier’s protocol. For each cell line 700,000 cells were centrifuged and frozen in RNAlater (Life Technologies, Darmstadt, Germany). RNA was extracted using the SPLIT RNA extraction kit (Lexogen GmbH, Vienna, Austria). Analysis of the large RNA fractions (> 150 nt) indicated very high RNA quality (RIN > 9.8 on an Agilent Technologies Bioanalyzer).

### mRNA-seq Library Preparation and Sequencing

Strand-specific NGS libraries were prepared from 500 ng RNA using the SENSE mRNA-Seq library preparation kit V1 (Lexogen GmbH, Vienna, Austria). For both cell lines, two different sized libraries were prepared. After the SENSE protocol size cut-offs, one library had an average size of 340 bp and the other one of 530 bp (assessed on an Agilent Technologies Bioanalyzer). The libraries were further limited in their size range by automated gel elution on a PippinPrep (Sage Science, Beverly, MA, USA). The shorter library was eluted with an average size of 299 bp (10% coefficient of variation, CV), the longer one with a size of 580 bp (8.6% CV). Subtracting the adapter sequence length, this translates into average insert sizes of 176 bp and 457 bp. A lane mix was prepared dedicating 40% of the final molar concentration to each of the two shorter libraries and 10% to each of the longer ones. Sequencing was performed on an Illumina HiSeq in paired-end 100 bp mode, and 285M PE reads were obtained. The read share analysis revealed that the longer libraries were represented at a lower degree than aimed for (see Experimental Validation below), possibly because of a more efficient cluster formation of the shorter libraries. Sequencing reads and the analysis results are available in NCBI Gene Expression Omnibus database (id:GSE56512).

### Experimental Validation Using qRT-PCR

Quantitative reverse transcription PCR was performed with 50 ng RNA using the Power SYBR^®^ Green RNA-to-CT^™^1-Step Kit (Life Technologies), according to the manufacturer’s protocol. All oligonucleotide primers except glyceraldehyde 3-phosphate dehydrogenase (GAPDH) were newly designed in this study and synthesized by Eurofins MWG Operon (Ebersberg, Germany). PCR efficiencies of the primers were validated using a standard method, and relative expression levels normalized to GAPDH was calculated by the comparative cycle threshold method. All reactions were performed in triplicate and results were plotted as average fold-change relative to GAPDH. Primer sequences can be found in the S1 Table.

## Results

### Simulated Datasets

Using our simulation pipeline and Ensembl 68 gene annotations, we created 50 RNA-seq datasets as described in the Materials and Methods. We ran InFusion along with five widely used tools for fusion detection—TopHat-Fusion, deFuse, ChimeraScan, SOAPfuse and fusionCatcher—on the generated RNA-seq datasets. We selected the first three tools for the assessment since they were reported to have high sensitivity and specificity in comparison to other tools [30] while the last two are quite novel and demonstrated the highest accuracy in a recent comparison study [45].

For each program we measured the number of true positive (*TP*) and false positive (*FP*) predictions among all discovered events, as well as recall $\left(\frac{TP}{\left(TP+FN\right)}\right)$ and precision $\left(\frac{TP}{\left(TP+FP\right)}\right)$. We considered a prediction as true positive if for each fusion partner the breakpoint position was reported within 20 bp upstream or downstream of the exact junction point defined by the simulation design. The number of false negative predictions was computed by analyzing how many simulated fusions were not detected in the dataset. In order to enable the discovery of a larger spectrum of fusion events and apply equal thresholds for fusion filtering, we configured the parameters of each tool accordingly (S1 Text). The results of the analysis are summarized in Table 1. InFusion demonstrated the best recall among the tested algorithms as well as a high level of precision. This precision level was similar to SOAPfuse and ChimeraScan, however InFusion was not losing the recall.

**Table 1:** Comparison of fusion detection tools on simulated data.

Tool	TP	FP	TOTAL	RECALL	PRECISION
InFusion	70 ± 4	5 ± 1	76 ± 4	0.70 ± 0.05	0.92 ± 0.02
ChimeraScan	38 ± 2	3 ± 1	42 ± 3	0.38 ± 0.03	0.92 ± 0.04
TopHat-Fusion	50 ± 4	20 ± 1	70 ± 5	0.50 ± 0.05	0.71 ± 0.02
deFuse	61 ± 6	30 ± 4	92 ± 7	0.63 ± 0.06	0.67 ± 0.04
SOAPFuse	55 ± 3	4 ± 2	60 ± 4	0.55 ± 0.04	0.92 ± 0.05
FusionCatcher	51 ± 4	17 ± 4	68 ± 6	0.51 ± 0.05	0.75 ± 0.05

Based on in silico data from 50 simulated RNA-seq datasets with 100 fusion genes each (5,000 fusions, ~10 million read pairs in total). N = total number of predictions reported by each tool, TP = true positives, FP = false positives. Each dataset was analyzed independently, and then mean value and standard deviation were computed for comparison.

**Fig 4:**
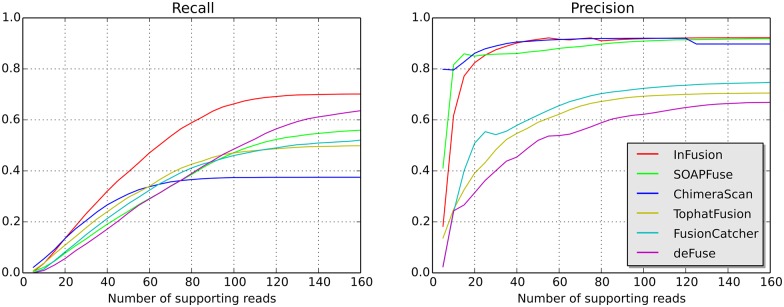
Comparison of recall and precision on simulated data. Recall and precision are plotted based on the number of supporting reads. For each given threshold we selected fusions that by simulation design have a number of supporting reads less or equal to the threshold. The number of true positive events was computed for every tool using only the selected fusions.

In order to investigate the effect of read coverage, we further analyzed recall and precision based on the number of reads supporting the fusion (Fig 4). InFusion demonstrated superior recall and high precision independent of the number of reads supporting the fusion event. Since the simulated transcripts consisted of five distinct classes, we also investigated the prediction accuracy for each fusion class independently (S1 Fig). For every class InFusion also achieved high recall and precision. It significantly outperformed other tools in detection of fusions that involve intronic and intergenic regions.

Additionally we performed special simulation experiments to validate the strand-specificity detection by InFusion. For this purpose certain reads simulating fusions were generated in 3 modes: forward strand-specific, reverse-strand specific and non-strand specific. As a result InFusion provided correct strand-specificity estimations for detected fusions. The detailed report can be found in S1 Text.

### Public Datasets

The performance of the InFusion pipeline was further tested by analyzing three public RNA-seq datasets from cancer studies: Edgren et al. [42] describe the transcriptome of cells originating from breast cancer, Berger et al. [43] from melanoma and Wu et al. [44] from prostate cancer. Each study provides evidence for fusion genes that were first detected from RNA-seq data and then experimentally validated using RT-PCR or Sanger sequencing. Reanalysis of the Edgren et al dataset performed by Kangapeska et al [34] enabled detection and validation of an additional 13 fusion events, which we also included in our test. We assessed the performance of the analyzed tools by comparing the number of rediscovered known fusions and the total number of fusions reported by each algorithm. For this comparison we used gene annotations from Ensembl version 68 and specific settings for each evaluated tool (S1 Text). In most cases no exact genomic locations for fusions were reported in these studies, therefore we considered a fusion event as rediscovered if both fusion partner genes were reported correctly. Results are summarized in Table 2.

**Table 2:** Fusion events detected in public RNA-seq datasets.

Dataset	Num. reads	Validated	InFusion	Chim.Scan	TopHat-Fusion	deFuse	SOAPFuse	FusionCatcher
Edgren et al	54.8 M	40	**37**|117	36|644	33|81	25|97	35|86	33|52
Berger et al	114.6 M	14	13|333	**14**|3733	6|17	11|277	13|159	2|6
Wu et al	253.4 M	26	**21**|373	14|2573	11|39	15|483	13|252	9|94

“Validated” refers to the number of fusions qRT-PCR-validated in the dataset as reported in the manuscript. For each fusion tool two values are provided. The first value is the number of previously validated fusions from the dataset also detected by a given tool, while the second value refers to the total number of fusions reported by the tool. Numbers in bold indicate the tool with the highest value of detected fusions for the corresponding dataset.

Overall, InFusion rediscovered the largest number of fusions reporting 71 events out of 80 previously described. In the dataset by Edgren et al 40 fusions were reported and InFusion achieved the highest detection rate, missing three known fusion events. One of them was not detected by any other tool, while two other fusions, reported by ChimeraScan, SOAPfuse and fusionCatcher, did not have sufficient number of reads to pass the filtering limits applied by InFusion. In the Berger et al dataset InFusion missed one known fusion event out of 14 reported. ChimeraScan demonstrated the highest detection rate, detecting the known fusion that was not discovered by either InFusion or any other of the tested algorithms. In this case the fusion was filtered out by InFusion due to its lack of reads spanning the corresponding junction. In the Wu et al dataset InFusion rediscovered the largest number of fusions (21 out 26 reported). Here InFusion filtered out five events because no unique reads covering the fusion junction were detected. These five fusions were also not detected by any other tool. The detailed list of fusion genes present in the datasets and their detection status can be found in S2–S4 Tables.

Higher numbers of reported fusions may be indicative of lower precision, although additional experiments are needed to estimate the actual proportion of spurious events. In our comparison, the number of non-validated fusions reported was lowest for Tophat-Fusion and FusionCatcher. InFusion reported a higher number of non-validated fusions, in the same range as SOAPFuse and deFuse, while ChimeraScan detects the highest number of non-validated events (Table 2).

It is also noteworthy that in the *Edgren et al* dataset InFusion along with fusionCatcher and TopHat-Fusion, reported isoforms of several known fusions that have been experimentally validated and described in detail [34]. Likewise in the *Berger et al* dataset InFusion detected the two distinct isoforms of fusion AXL–REC, reported by the authors [42]. Both isoforms were also discovered by deFuse and fusionCatcher.

### Experimental Validation

To further investigate the power of the InFusion pipeline to detect chimeric transcripts from deep sequencing data, we sequenced the mRNA of two well-established prostate cancer cell lines, VCaP and LNCaP. Both cell lines are known to harbour genomic translocations and well-studied fusion genes [18]. Using the strand-specific SENSE mRNA library preparation kit (Lexogen GmbH, Vienna, Austria), we constructed two libraries for each cell line with average insert sizes of 176 bp (referred to as VCap200 and LNCaP200) and 457 bp (referred to as VCap500 and LNCaP500). Sample allocation and detailed sequencing statistics are shown in Table 3.

**Table 3:** RNA-Seq sample details.

Cell line	Sample	*Mean insert size (bp)*	*Total read pairs*
VCaP	VCaP200	176	71,229,410
VCaP	VCaP500	457	7,653,307
LNCaP	LNCaP200	176	74,575,800
LNCaP	LNCaP500	457	4,521,899

List of samples with associated number of reads obtained from the deep sequencing of VCaP and LNCaP cell lines

We ran the InFusion pipeline on the datasets VCaP200 and LNCaP200 and detected 336 and 338 putative fusion events, respectively. The number of detected fusions stratified by fusion type can be found in S5 Table. For the experimental validation, aiming to cover the whole spectrum of fusion events, 21 candidates from VCaP and 19 from LNCaP were selected and subjected to qRT-PCR (Table 4). Ten out of these 40 were known fusions [18] that we used as controls, while the others were novel and to the best of our knowledge have not been reported previously. Four events were selected as indicating anti-sense transcription and nine events as fusions of a coding with an intergenic or intronic region. Four selected events were isoforms of known fusions. The remaining 13 novel events were chosen randomly for validation.

**Table 4:** Fusions selected for qRT-PCR validation from RNA-seq of VCaP and LNCaP cell lines.

*Name*	*Chr1*	*Pos1*	*Break1*	*Chr2*	*Pos2*	*Break2*	*Split reads*	*Bridge reads*	*Cell line*	*qPCR*
SREBF2—XRCC6	22	42271728	Exon edge	22	42032115	Exon edge	587	0	LNCaP	+
VWA2 –PRKCH	10	116008524	Exon edge	14	61909827	Exon edge	109	0	VCaP	+
INSL6—JAK2	9	51641179	Exon edge	9	49992434	Inside intron	87	3	VCaP	+
ZDHHC7—H3F3B	16	85029526	Exon edge	17	73775267	Exon edge	45	36	VCaP	+
ZDHHC7—UNK I1	16	85029528	Exon edge	17	73782537	Inside intron	53	27	VCaP	+
**HJURP—EIF4E2**	2	234749255	Exon edge	2	233421124	Exon edge	59	12	VCaP	+
**FAM117B - BMPR2**	2	203500510	Exon edge	2	203329530	Exon edge	56	0	LNCap	+
**GPS2—MPP2**	17	7218278	Exon edge	17	419757748	Exon edge	51	5	LNCaP	+
Intergenic—NBEA	13	35692611	Intergenic	15	20851765	Exon edge	44	0	Both	+
**SLC45A3—ELK4 I1**	1	205630992	Exon edge	1	205593020	Exon edge	41	0	LNCaP	+
PPIP5K2—CTC-340A15.2	5	102465407	Exon edge	5	164598384	Exon edge	11	26	VCaP	+
**RC3H2—RGS3 I1**	9	125622199	Exon edge	9	116299073	Exon edge	32	2	VCaP	+
AAK1—AC114772.1	2	69732701	Exon edge	2	69693684	Inside exon	33	0	Both	+
ZDHHC7—UNK I2	16	85027706	Inside intron	17	73782537	Inside intron	26	0	VCaP	+
ZNF577—ZNF841	19	52380532	Exon edge	19	52570866	Exon edge	24	1	VCaP	+
CTA-221G9.11—KIAA1671	22	25508430	Inside exon	22	25566787	Exon edge	22	0	Both	+
**TMPRSS2—ERG I1**	21	42879876	Exon edge	21	39817547	Exon edge	20	0	VCaP	+
RP11-534G20.3 –SVIL	10	29704341	Exon edge	10	29746577	Inside exon	20	0	LNCaP	+
SPOCK1 –Intergenic	5	136602698	Exon edge	5	180144798	Intergenic	15	3	VCaP	+
POLR1D - LNX2	13	28195176	Inside exon	13	28155942	Exon edge	18	0	Both	+
HSF1 –RERE	8	145515556	Exon edge	1	8716501	Exon edge	16	0	VCaP	+
**MIPOL1 –DGKB**	14	37969347	Inside exon	7	14188860	Exon edge	16	0	LNCaP	+
Intergenic—SH3D19	4	152246392	Intergenic	4	152147395	Exon edge	15	0	VCaP	+
**TIA1—DIRC2**	3	122552164	Exon edge	2	70475537	Exon edge	12	0	VCaP	+
TMPRSS2—ERG I2	21	42879876	Exon edge	21	39846047	Exon edge	11	0	VCaP	+
CNNM4—PARD3B	2	97474487	Inside exon	2	205829873	Exon edge	11	0	VCaP	+
SLC45A3—ELK4 I2	1	205628618	Inside exon	1	205593020	Exon edge	10	1	LNCaP	+
RC3H2—RGS3 I2	9	125621318	Inside exon	9	116299074	Exon edge	8	1	VCaP	+
AC024940.1—FAM60A	12	31477418	Inside exon	12	31451159	Exon edge	9	0	VCaP	+
**RERE—PIK3CD**	1	8482786	Exon edge	1	9770482	Exon edge	9	0	LNCaP	+
RP11-180P8.1—TANC2	17	61044108	Inside exon	17	61086895	Inside exon	9	0	Both	+
TMED2 –INTERGENIC	12	124082643	Inside exon	6	79645813	Intergenic	8	0	LNCap	-
SEC22B - AKT1	14	105237110	Inside exon	1	145116734	Inside exon	7	0	LNCap	-
Intergenic—AMZ2	17	66202379	Intergenic	17	66246327	Exon edge	6	0	LNCap	+
RAD9A - UBQLN2	11	67163530	Inside exon	X	5690882	Inside exon	2	4	LNCap	-
TMPRSS2—ERG I3	21	42880007	Exon edge	21	39817547	Exon edge	5	0	VCaP	+
DIRC2 –Intergenic	3	122545912	Exon edge	2	64697744	Intergenic	3	2	VCaP	+
CASZ1 –KAZN	1	10820755	Exon edge	1	15070470	Inside exon	5	0	LNCap	+
ST7—DSG2	7	29128146	Inside exon	18	116869869	Inside exon	5	0	LNCap	-
**LMAN2—AP3S1**	5	176778447	Inside exon	5	115202365	Exon edge	2	1	VCaP	+

Fusions in bold have been previously reported and were used as controls. In column “Cell line” “Both” indicates that the fusion was confirmed both in VCaP and LNCaP.

The qRT-PCR confirmed 36 out of the 40 selected chimeric transcripts, including all ten control fusions (Table 4). Five out of 26 novel events were validated in both cell lines, while the remaining 21 were specific for one cell line only. Interestingly, all the events verified in both cell lines appear to be intra-chromosomal with the exception of a single chimeric transcript that involves gene NBEA on chromosome 15 and an intergenic region on chromosome 13. A further four (validated) predictions with an intergenic region as a second fusion partner were detected only in one cell line. Fusions INSL6–JAK2 and two isoforms of ZDHHC7–UNK present in the VCaP cell line have a breakpoint inside the intron of the 5’ fusion gene partner. Notably, four confirmed events (POLR1D–LNX2, CTA-221G9.11–KIAA1671, CTC-340A15.2–PPIP5K2, RP11-534G20.3–SVIL) indicate antisense transcription, emphasizing the value of a strand-specific library preparation (for more details see S1 Text).

To investigate the effect of the NGS library insert size on fusion detection, we analyzed datasets VCaP500 and LNCap500. These samples received only 9% and 5% of the reads of their VCaP200 and LNCap200 counterparts, respectively. Strikingly, InFusion revealed 151 putative fusions in the VCaP500 sample and 87 in the LNCaP500 sample and moreover, we observed that only 15 and 9 fusions, respectively, were shared between the libraries with short and long inserts. The majority of fusion predictions are exclusive to either small or large insert size libraries (S2 Fig). Fourteen PCR-validated fusions were also found in datasets with larger insert size and lower coverage (S3 Fig), and by qRT-PCR we additionally confirmed one novel isoform of fusion SLC45A3–ELK4, which was found only in the sample LNCaP500.

**Fig 5:**
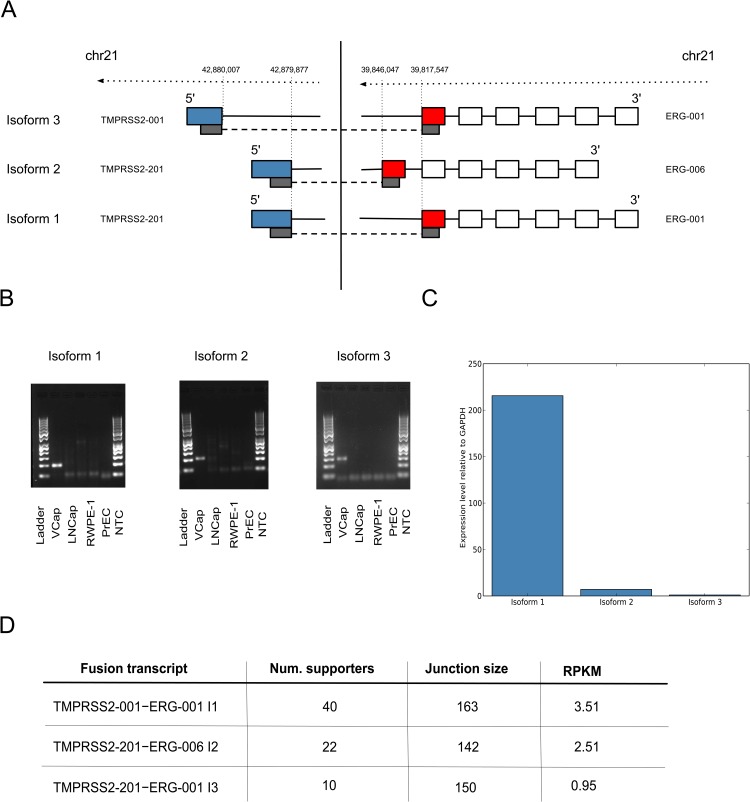
TMPRSS2-ERG fusion isoforms. (A) Genomic structure of the TMPRSS2–ERG fusion transcripts discovered from deep sequencing data by InFusion. Isoform 3 is a known transcript, while isoforms 1 and 2 are novel. Transcript names are taken from the Ensembl v.68 database. (B) RT-PCR validation of isoforms in VCaP, LNCaP, RWPE-1 and PrEC cell lines; NTC = no template control. The PCR primer design was based on the output from the InFusion pipeline. In order to detect only one product, one PCR primer specific for Isoform 3 was designed to cover the fusion junction site. A 50 bp DNA ladder was co-run as size marker; bright bands indicate 250 bp and 500 bp. (C) Relative expression levels of the fusion isoforms as measured by qRT-PCR. All measurements were performed in triplicate, mean expression values were computed relative to GAPDH. Plotted values are normalized to the computed expression of isoform 3. (D) Expression levels of isoforms estimated in RPKM under the assumption of uniform coverage.

Interestingly, we detected and verified several novel splice variants of the known fusions TMPRSS2–ERG, RC3H2–RGS3 and SLC45A3–ELK4. Three isoforms of TMPRSS2–ERG were also tested by qRT-PCR in the non-cancerous prostate cell lines RWPE-1 and PrEC, but there was no evidence of these transcripts in cell lines other than VCaP (Fig 5A and 5B). Overall, both in qRT-PCR and expression analysis isoform 1 has the highest expression compared to isoforms 2 and 3, however in qRT-PCR it dominates significantly over other isoforms (Fig 5C and 5D). Surprisingly, isoform 3, which is the best described isoform, has the lowest expression level, as measured by qRT-PCR, and the least support from the sequencing data compared to other isoforms.

Another intriguing observation concerns fusions that involve intergenic regions, which are typically ignored by other fusion detection tools. We discovered that they constitute a large proportion of the predictions. For example 94 out of 336 predictions in VCaP200 sample and 95 out of 338 predictions in LNCaP200 sample have an intergenic region as one part of a fusion. Similar proportions of fusions involving intergenic regions were also found in public datasets (S6 Table). A curious example of such an event is a validated inter-chromosomal fusion in the VCaP cell line that connects the DIRC2 gene and an intergenic region of chromosome 2, a fusion that is supported by a large clustering of reads downstream of the predicted breakpoint (S4 Fig).

Additionally, we analyzed the cohort of experimentally validated fusions with TopHat-Fusion, deFuse, ChimeraScan SOAPfuse and fusionCatcher, as we did in previous comparisons (S7 Table). While a number of novel fusion events was also detected by other tools, 14 novel fusions including several isoforms and events involving intronic and intergenic regions were detected only by InFusion. Certain isoforms of TMPRSS2-ERG and RC3H2-RGS3 were also detected by fusionCatcher and SOAPfuse, and two intronic fusions ZDHHC7—UNK by TopHat-Fusion and deFuse. However, InFusion outperformed these tools in the total detection of these classes.

## Discussion

We have designed and implemented a novel method for chimeric transcript discovery from RNA-seq data. Our method combines and improves ideas proposed by other researchers, such as assignment of reads that map to multiple loci and advanced fusion filtering. Additionally, it introduces several novel algorithmic aspects of chimeric RNA discovery, including intergenic region analysis, fusion cluster homogeneity estimation and consideration of protocol strand specificity. Our comparative analysis demonstrates that InFusion outperforms existing approaches for chimeric transcript discovery in recall and has adjustable detection accuracy.

Using simulation data, we show that InFusion is able to discover a wide spectrum of fusion events that can occur in the transcriptome. Importantly, from our experimental data we discovered in-silico and verified in-vitro alternatively spliced fusion isoforms and chimeric RNAs involving non-exonic regions. In concordance with recent studies [46] we observed that in most cases of fusion genes one transcript isoform is dominant and highly expressed, while the other isoforms are transcribed at significantly lower levels. However, this expression pattern may be completely different at a different time point or in another cell type, and isoforms might encode for RNAs or proteins of different functionality, which makes isoform detection important for differential gene expression analysis.

Remarkably, in our predictions from cancer data we observed a large number of fusions that involve intergenic regions, and four tested fusions of this kind were confirmed in vitro. To our knowledge, discovery of such events has not been addressed previously, despite their potential to encode functional proteins or regulate gene transcription.

An important factor influencing the detection of fusions from RNA-seq data is the depth of coverage of the sequencing experiment. Similar to novel transcript discovery and alternative splicing studies, fusion gene discovery benefits from higher coverage depth and longer reads [47]. Our analysis shows that highly expressed fusions can be revealed even with relatively low coverage (S5 Fig), however, a gain in sequencing throughput greatly increases the sensitivity of discovery. Additionally, it is advantageous to use sequencing libraries with various insert sizes, since the fragment length affects the range of detectable fusion events in paired-end sequencing. A notable example of this effect is the additional validated isoform of fusion SLC45A3–ELK4, which we detected only in the low-covered sample LNCaP500. Furthermore, strand-specific protocols are preferable for fusion discovery since they allow analysis of antisense transcription [48]. InFusion uses information from the strand specificity of the library to report antisense transcription in chimeric RNAs and also infers the transcription strand in case of a non-annotated region. The computational efficiency of InFusion allows it to process large RNA-seq datasets comparatively quickly, e.g. it took approximately 10 h to analyze 74 million 100 bp paired reads on a machine with eight 2.4 GHz CPUs and the memory requirements did not exceed 30 GB.

There are several directions for future work. Firstly, how exactly read size benefits fusion detection remains to be determined. We performed additional simulation experiments to check the influence of read size (S6 Fig). We found that in comparison to existing tools InFusion maintains high precision, however, recall decreases with increase of read size in all tools due to a growing number of break breakpoints in SPLIT reads. We plan to improve the recall for larger read size in future versions of the program. Second, estimating fusion gene expression remains an open problem. InFusion partly solves this issue by allowing output of possible fusion transcript sequences, which can be added to the transcriptome library so that expression can be quantified by applying methods such as RSEM [49]. However, we believe more robust solutions might be possible. Third, the discovery of the fusion origin cannot be addressed using RNA-seq short reads alone. In order to determine if the chimeric transcript originates from a genomic translocation, from trans-splicing or from an experimental artefact, transcriptome sequencing should be combined with other experimental technologies. There are methods that combine long and short RNA-seq reads [50] or whole genome sequencing and transcriptome sequencing data to detect gene fusions [51], but more work is required in this area. Correlating fusions with cancer will continue to provide new insights into this disease and inform personalized therapy [28]. Chimeric transcripts, on the other hand, have also been shown to occur in non-cancerous cells due to trans- or cis-splicing [29, 52], but the underlying mechanisms have yet to be studied in depth [28]. InFusion may prove to be a useful tool with high software quality in furthering our understanding in this area by detecting the whole scope of possible events.

## Supporting Information

S1 Text Supplementary materials including a description of the simulation pipeline, parameters applied for the fusion discovery tools in computational experiments and detection of sense-antisense chimeric transcripts from RNA-seq data. (DOCX)

S1 Fig Comparison of recall and precision on simulation data per fusion class. Simulated fusions and corresponding datasets were analyzed per fusion class: (A) with both fusion partner(s) having a breakpoint at the exon boundary; (B) with one or both fusion partner(s) having a breakpoint inside an exon; (C) with one or both fusion partner having a breakpoint inside an intron (D) with several alternatively spliced isoforms (E) with one fusion partner originating from an intergenic region.
(EPS)

S2 Fig Effect of insert size on fusion discovery. The figure shows comparison of fusion predictions for samples with different insert sizes in the same cell line. The lower number of fusions discovered reflects the lower sequencing depth in VCaP500 (9.2% of VCap200) and LNCaP500 (5.2% of LNCaP200).
(EPS)

S3 Fig PCR-validated fusions detected in the sequenced samples. (A) VCaP cell line (B) LNCap cell line. Previously described fusions are shown in bold. Fusions detected in both cell lines are shown in italics.
(EPS)

S4 Fig Screenshots from IGV demonstrating alignment of reads for a confirmed fusion “DIRC2 –intergenic” (A) 5' part of the fusion, the breakpoint is on the exon boundary of gene DIRC2 (B) 3' part of the fusion, the breakpoint is in the intergenic region on chromosome 2. The alignments shown in light red are supporting the fusion junction. The red vertical line denotes fusion breakpoint, while the arrow shows direction of transcription.
(EPS)

S5 Fig Fusion detection performed from subsamples. The subsamples are generated from (A) VCaP200 and (B) LNCaP200 RNA-seq datasets. Each subsample is created randomly from the dataset based on the required size. The sample size changes from 5 M reads to 70 M with a step of 5 M.
(EPS)

S6 Fig Read size influence on recall and precision in fusion detection. Fusion simulation was performed for read size from 75bp to 200 bp (10 samples for each experiment). Then fusion detection was applied by InFusion along with SOAPfuse and fusionCatcher. Recall (A) and precision (B) in fusion detection were computed for the tool results.
(EPS)

S1 Table qRT-PCR primer pairs. Primer pairs used for qRT-PCR validation of the 40 selected fusion genes discovered from the deep sequencing data of VCaP and LNCap cell lines. 4 fusions (in bold) were not validated. This issue might be due to the generation of false artificial chimeras during the reverse transcriptase step in RNA-sequencing or a qRT-PCR-related problem. Notably, 6 additional qRT-PCR experiments were initially performed for fusion events that were reported from an earlier InFusion version which were not verified. Subsequent improved versions of InFusion do not report those events.
(XLSX)

S2 Table Fusion genes in the Edgren et. al dataset. Showing whether validated fusions were detected by selected tools. Additionally the table includes fusions detected and validated by Kangapeska et al. [34] (in italics).
(XLSX)

S3 Table Fusion genes in the Berger et. al dataset. Showing whether validated fusions were detected by selected tools.
(XLSX)

S4 Table Fusion genes in the Wu et. al dataset. Showing whether validated fusions were detected by selected tools.
(XLSX)

S5 Table Fusion types detected in VCap and LNCaP cell lines. **Total** is the total number of fusions detected. **Exon boundary break** is the number of fusions where both 5' and 3' fusion breaks are on the exon boundary. **With isoforms** is the number of fusions of the same type that have several isoforms. **Break inside exon** is the number of fusions where one or both breaks are inside an exon. **Break inside intron** is the number of fusions where one or both breaks are inside an intron. **Within intergenic** is the number of fusions where one of the breaks is inside an intergenic region.
(XLSX)

S6 Table Analysis of public datasets with support of fusions within intergenic region. Public datsets Edgren et al [42], Berger et al [43] and Wu et al [44] were reanalysed by InFusion with the option to report fusions with one break inside an intergenic region activated. The table provides novel results for each sample of the datasets.
(XLSX)

S7 Table Fusions from VCaP and LNCaP cell lines. The table indicates if a particular event detected by InFusion and validated using qRT-PCR was also reported by tools deFuse, TopHat-Fusion, ChimeraScan, SOAPfuse and fusionCatcher. Fusions in bold are detected and reported only by InFusion.
(XLSX)
