# Supplementary material for: InFusion: Advancing Discovery of Fusion Genes and Chimeric Transcripts from Deep RNA-Sequencing Data
Source: PLoS One. 2016 Dec 1;11(12):e0167417. doi: 10.1371/journal.pone.0167417 (PMC5132003; doi:10.1371/journal.pone.0167417)
Supplement: S1 Text — (DOCX) [file pone.0167417.s001.docx]

***Okonechnikov K. et al.***

**InFusion: advancing discovery of fusion genes and chimeric transcripts from deep RNA-sequencing data**

**Supplementary materials**

**Fusion simulation: data generation and analysis**

The fusion simulation and analysis pipeline is available as a part of the InFusion toolkit source code.

The base procedure of the pipeline is as follows:

1. Create the fusions of a specific type
2. Generate transcript sequences forming the fusions
3. Simulate reads supporting the fusion transcripts and combine them with background reads based on selected coverage settings
4. Launch the analysis of fusion discovery tools on the generated datasets
5. Compute specific statistics from the results reported by the selected tools, e.g. recall, precision, f-measure, breakpoint position accuracy etc.

In our simulation procedures we applied background reads generated based on the sample SKBR3 from the Edgren et al dataset [42] (breast cancer RNA-seq data). To perform this task the expression level of the sample was computed by performing the alignment and computing read counts. Afterwards, the background reads were generated based on the proportion from reported counts. The total number of selected transcripts was 117156. Additionally, the expressed genes were applied to form fusion groups based on a known exon boundary, while other fusion types were generated randomly. The total number of generated background reads was 31398226.

In the comparison of existing fusion discovery tools we also investigated the precision of breakpoint position computed by the tools for reported valid fusions. To perform this task from 50 analyzed simulation datasets we computed the proportion of valid fusions reported with exact junction position and the mean shift from the correct breakpoint for all reported fusions:

| Tool | Number of valid fusions reported | Exact junction proportion | Mean breakpoint  shift (bp) |
| --- | --- | --- | --- |
| **InFusion** | 6728 | 0.89 | 0.18 |
| SOAPFuse | 4988 | 0.91 | 0.12 |
| FusionCatcher | 4074 | 0.62 | 0.67 |
| defuse | 6294 | 0.88 | 0.17 |
| ChimeraScan | 1422 | 0.92 | 0.72 |
| TopHat-Fusion | 4962 | 0.91 | 0.14 |

Most of the tools, including InFusion, were quite precise in reporting the correct breakpoint position. However, ChimeraScan and FusionCatcher were showing quite high shifts (up to 20 bp). Based on this investigation, the default breakpoint position shift was selected.

**Fusion discovery tools options**

This section describes parameters applied for the fusion discovery tools in computational experiments.

**General**

In our comparison we analyzed output of **InFusion v0.7.2** (files: *fusions.txt*), **Chimerascan v.0.4.5** (file: *chimeras.bedpe*), **deFuse v0.6.0** (file: *results.filtered.tsv*), **TopHat-Fusion v2.0.2** (file: *result.txt*), **SOAPfuse v1.26** (file: *soap_sample.final.Fusion.specific.for.genes*) and **fusionCatcher v0.99.4a,b** (file: *final-list_candidate-fusion-genes.GRCh37.txt*).

All described tools require genome and transcriptome sequences as well as gene annotations. For each tool we used hg19 genome build. We applied Ensembl v.68 gene annotations for InFusion, SOAPfuse and deFuse, while TopHat-Fusion, Chimerascan and fusionCatcher used internal custom-formatted annotation datasets based on Ensembl and UCSC RefSeq databases.

**Simulated data analysis**

In order to increase the spectrum of detected events we applied specific options for analyzed tools. Additionally, we set the same thresholds for the numbers of supporting SPLIT and BRIDGE reads where possible.

**InFusion**

We enabled the detection of non-coding and intergenic regions:

*infusion --allow-non-coding --allow-intronic --allow-intergenic -1 $READS_1 -2 $READS_2*

*/data/fusions-data/infusion.ens68.cfg*

**ChimeraScan**

In ChimeraScan it was not possible to set a minimum threshold for the number of supporting reads in the configuration, therefore we applied additional filtering of the results to establish the limits for 1 split and 4 span supporting reads.

**deFuse**

We changed the minimum required number of supporting split and span segments in the configuration to 1 and 4 accordingly, which corresponds to the default InFusion values:

*#*

*# Configuration file for deFuse*

*#*

*span_count_threshold = 4*

*split_count_threshold = 1*

**TopHat-Fusion**

When running TopHat we set the minimum fusion distance to 20000, which corresponds to the default value of a similar InFusion parameter {max-intron-size}. Other parameters were set as recommended by the authors of the program in the **“Getting Started"** tutorial:

*tophat -o tophat_simulation -p 4 --fusion-search --keep-fasta-order*

*--bowtie1 --no-coverage-search -r 100 --mate-std-dev 300 --fusion-min-dist 20000*

*/data/tophatruns/index/hg19 $READS_1 $READS_2*

In the *tophat-fusion-post* script we set the thresholds for supporting reads which correspond to the defaults in InFusion:

*tophat-fusion-post -p 4 --num-fusion-both 4 --num-fusion-reads 1*

*--num-fusion-pairs 0 $TH_DATA/index/hg19*

**SOAPfuse**

In the main settings of SOAPfuse we did not change any requirements. However the required correct insert size in the configuration file for each experiment was provided.

**fusionCatcher**

We used default configuration parameters for fusionCatcher since it adapts correctly to the provided data without additional settings.

The pipeline depends on a certain number of tools with fixed version. Unfortunately, certain bugs were introduced by STAR aligner as one of the included tools; however automatic ***restart.sh*** script was used to rerun analysis of failed experiments. Additionally, the novel version of fusionCatcher (0.99.4b) relies on a new version of STAR, and these problems were not seen again.

**Public datasets**

For the public datasets, the settings of InFusion were adapted to provide optimal results. Also, the option to discover fusions including intergenic regions was not activated initially to allow comparison of the results with other benchmarked tools. An additional analysis was performed to investigate the proportion of fusions with intergenic regions.

**Edgren et al** [42]

BT474:

*--allow-non-coding --allow-intronic --do-coverage-analysis --min-unique-split-reads 0 --min-bckg-reads 2 --req-homogeneity-weight 0.15*

KPL4:

*--allow-intronic --do-coverage-analysis --min-unique-split-reads 0 --min-bckg-reads 2*

MCF7:

*--allow-intronic --allow-non-coding --do-coverage-analysis --min-split-reads 0 --min-unique-split-reads 0 --min-bckg-reads 2*

SKBR3:

*--allow-intronic --do-coverage-analysis --min-bckg-reads 2*

**Berger et al** [43]

K562:

*--allow-intronic --do-coverage-analysis --min-novel-junc-split-reads 1*

M000921:

*--allow-intronic --do-coverage-analysis --min-unique-split-reads 0*

All other samples:

*--allow-intronic --do-coverage-analysis*

**Wu et al** [44]

LNCAP:

*--allow-non-coding --allow-intronic --do-coverage-analysis --min-unique-split-reads 0 --min-bckg-reads 3*

LTL313H:

*--allow-intronic --do-coverage-analysis --min-bckg-reads 3*

For other tools we used the results provided in the corresponding publications if the full report including total number of reported fusions was available (TopHat-Fusion for Edgren et al [42] dataset). In the remaining cases the same parameters as in the simulation experiment were applied and optimized to reach the highest recall of validated fusions when it was possible.

**In-house datasets**

We applied the following configuration for analysis of deep sequencing data from VCap and LNCap cell lines:

*infusion --library RF –allow-intronic --allow-intergenic --allow-non-coding --min-split-reads 2 --min-span-pairs 0 --min-fragments 3 --min-unique-split-reads 0 --min-unique-alignment-rate 0.04*

*-1 $READS_1 -2 $READS_2 /data/fusions-data/infusion.ens68.cfg*

**InFusion results filtering control**

The InFusion filtering procedure depends on multiple parameters and can be controlled to keep the balance between sensitivity and specificity. The fusion score is formed as a combination of multiple mark-factors. For each factor there is a specific filtering limit. The main mark-factors for the fusion candidate are the supporting reads with various properties.

The following filters allow control of the limits:

- Minimum number of SPLIT read alignments
- Minimum number of BRIDGE read pairs
- In case of paired-end data: minimum sum of SPLIT reads and BRIDGE pairs supporting the fusion
- Minimum number of unique alignments forming the SPLIT reads
- Minimum number of SPLIT reads supporting a novel junction site
- Minimum number of SPLIT reads not rescued

Additionally, it is possible to use the weight variable (default is 1) for SPLIT-read or BRIDGE pair to adjust the filtering. Multiple other filters are related to other properties, such as minimum weight of the fusion in a “metacluster” (combination of overlapping clusters) or permission to allow fusions within intronic/intergenic regions.

By default the InFusion options, such as minimum number of SPLIT and BRIDGE reads, are adapted for high specificity. To get the list of confident fusions, it is also possible to discard fusions that are supported only with multimapped reads. Finally, a higher threshold for the number of supporting fragments can be applied.

To increase the sensitivity, it is beneficial to allow intronic, intergenic and non-coding segments for fusion forming. Additionally, the thresholds for the number of supporting fragments can be lowered. The limits depend on the total number of reads in the sequencing procedure and can be adjusted by running only the filtering procedure several times with different settings.

**Impact of strand-specificity on detection of fusions from RNA-seq data**

This section describes the support of strand-specificity by the InFusion toolkit. The results from simulation data and real dataset are provided and described in detail.

**Influence of strand-specificity**

The strand-specific sequencing library provides the following advantageous features:

1) Detection of antisense transcription in fusions involving known genes

2) Inference of the direction of transcription in fusions involving un-annotated and intergenic regions

3) Differentiation between fusion isoforms that involve anti-sense transcription

Strand-specificity provides a deeper insight into the structure of chimeric transcripts. It does not influence the fusion discovery in general, but rather raises the resolution of the analysis and provides additional important information about the fusions.

The absence of strand-specificity may affect fusion discovery only in specific cases. One example is the differentiation between two isoforms of the same gene fusion, one of which is being transcribed antisense. Please see figure “Strand-specificity and fusion isoforms” below for illustration of such an event.


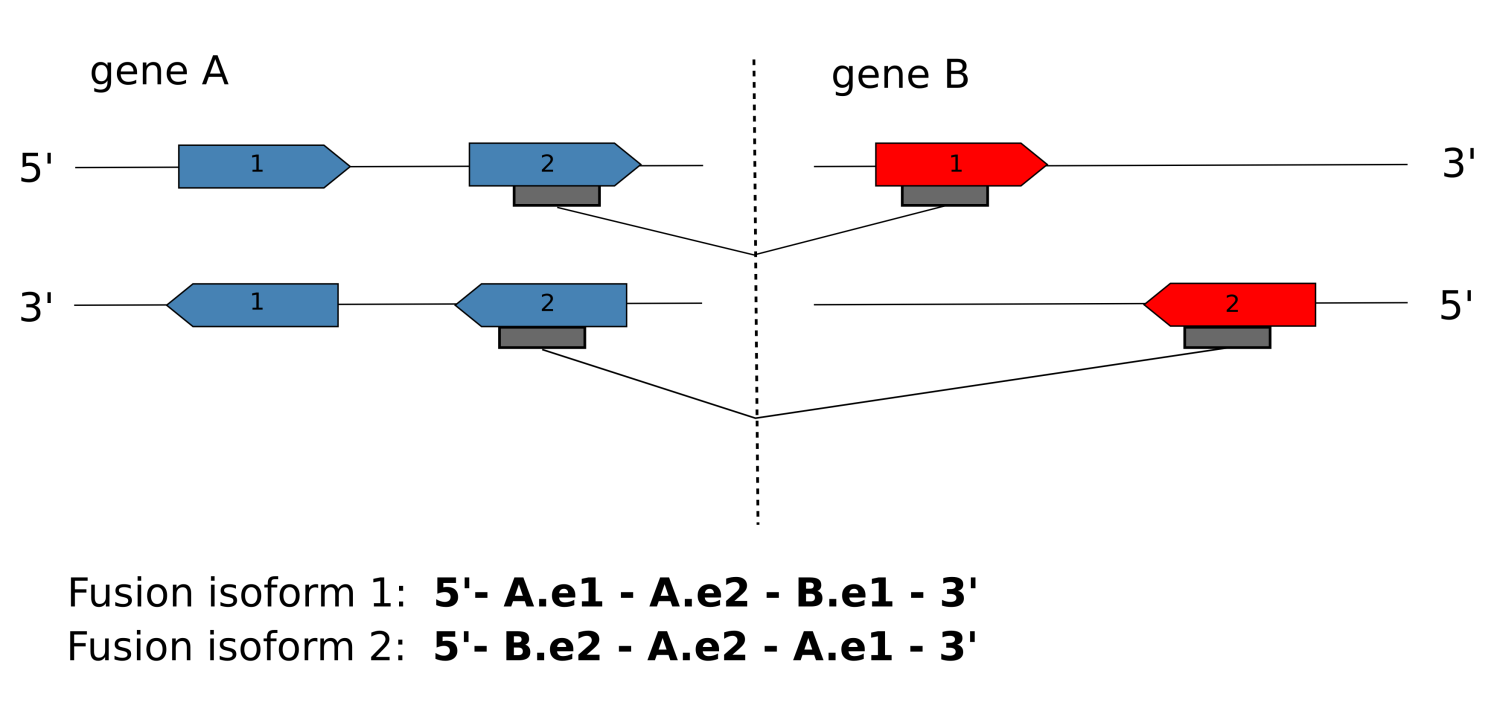


**Figure “Strand-specificity and fusion isoforms”.** Let us assume that there are 2 isoforms of a fusion involving genes A and B. The first isoform involves exons 1, 2 of gene A and exon 1 of gene B. It is being transcribed in forward strand, which is a sense strand for genes A and B. The second fusion involves exon 2 of gene B and exons 2,1 of gene A. It is being transcribed anti-sense, in reverse complement strand. Since there are only paired BRIDGE-reads (colored in gray) supporting both fusions, the correct configuration can be inferred only by taking into account strand-specificity.

**Detecting sense-antisense chimeras by InFusion**

To be consistent with the available public datasets and also to perform a fair comparison to other methods, all simulation experiments discussed in the manuscript were performed without strandness.

To assess the capability of InFusion to work with strand-specific data we performed additional simulation experiments. We asked the following questions:

1) Does InFusion process the strand-specific data correctly?

2) Is the pipeline capable of discovering antisense transcription in fusions and infer correct transcription direction?

To answer these questions, we performed fusion simulation runs using three strand-specific protocols: forward-strand-specific, non-strand-specific and reverse-strand specific. Additionally for each simulation run we provided background read data in non-strand specific mode. Each simulation was performed 3 times. Then we applied InFusion and checked if the resulting fusion transcript strand-specificity was detected correctly according to the protocol. The results are reported in the table “Mean strand specificity proportion detected by InFusion”.

| **Simulated fusion**  **strand specificity type** | **Computed SSP value without/with background non-strand-specific reads** |
| --- | --- |
| Reverse-strand-specific | 0.035475 / 0.035477 |
| Non-strand-specific | 0.499587 / 0.499436 |
| Forward-strand-specific | 0.970641 / 0.970969 |

**Table “Mean strand specificity proportion detected by InFusion”.** InFusion reports strand-specificity proportion (SSP) for each fusion. SSP validates from 0 (reverse-strand speficic) to 1 (forward-strand-specific). For each dataset mean detected SSP among all fusions was computed.

The simulation results demonstrate that InFusion provides the correct strand-specificity proportion for detected fusions regardless of the type of background reads strand-specificity.

**Sense-antisense chimeras detected in VCaP and LNCaP cell lines**

Additionally we checked and analyzed in detail antisense chimeras detected from VCAP and LNCaP cell-lines. Overall we confirmed 4 events detected by InFusion that indicate antisense transcription from supporting reads:

1. **POLR1D (+): LNX2 (-)** [Both VCap and LNCap]

This fusion transcript connects the first exon of POLR1D gene transcribed in antisense direction with the second exon of the LNX2 gene. This is most likely a read-through event (transcription of bordering/intersecting genes) or a trans-splicing product since it is was validated in both cell-lines and genes are adjacent. Alternatively this could be an isoform of LNX2 gene, which intersects POLR1D.

2. **PPIP5K2 (+)** - **CTC-340A15.2 (+)** [VCap]

This chimeric transcript connects the second exon of PPIP5K2 with the last exon of an Ensembl-annotated construct CTC-340A15.2, which is known to be transcribed antisense to a pseudogene CTC-340A15.1. The strand specificity analysis indicates that the 3’ part of the fusion is being transcribed from the forward strand, which matches the strand of the PPIP5K2 gene. This fusion is interesting from a functional point of view, because it disrupts the ORF of PPIP5K2 and most likely originates from a genomic translocation.

3. **CTA-221G9.11 (-): KIAA1671 (+)** [LNCap]

This chimeric transcript connects a region that belongs to an Ensembl-annotated antisense RNA CTA-221G9.11 with an exon of the KIAA1671 gene. The 5’ part of the transcript additionally intersects the intron of KIAA1671 and has the same transcription strand, thus this could be an unknown exon of this gene. The examination of transcriptome alignment showed that there are fragments aligned to CTA-221G9.11 in both strands (proportion 13:33), which indicates the presence of sense and antisense transcription.

4. **RP11-534G20.3 (PCTH3DP1) (+) - SVIL (-)** [LNCaP]

This is most likely a read-through event. It connects one of the exons of the RP11-534G20.3 gene with the first exon of an adjacent SVIL-1 gene, but is transcribed in the antisense direction (forward strand).
